# Portal Vein Thrombosis after C-Section in a Patient with Polycythemia Vera (PV) Due to Pregnancy and Iron Deficiency Anemia (IDA)

**DOI:** 10.3390/clinpract12060109

**Published:** 2022-12-14

**Authors:** Thomas Ntounis, Konstantinos A. Zioutos, Antonios Koutras, Ioannis Prokopakis, Zacharias Fasoulakis, Ioakeim Sapantzoglou, Paraskevas Perros, Athina A. Samara, Emmanouil Spanoudakis, Asimina Valsamaki, Sevasti-Effraimia Krouskou, Konstantinos Nikolettos, Vasileios-Chrysovalantis Palios, Paschalis Mousios, Kallirroi Goula, Kyriakos Konis, Athanasios Chionis, Emmanuel N. Kontomanolis

**Affiliations:** 1 1st Department of Obstetrics and Gynecology, National and Kapodistrian University of Athens, General Hospital of Athens ‘ALEXANDRA’, Lourou and Vasilissis Sofias Ave, 11528 Athens, Greece; 2 Department of Obstetrics and Gynecology-Kastoria General Hospital, Mavriotissis 33, 52100 Kastoria, Greece; 3 Department of Embryology, University General Hospital of Larissa, Mezourlo, 41110 Larissa, Greece; 4 Department of Hematology, Democritus University of Thrace, University General Hospital of Alexandroupolis, 6th km Alexandroupolis–Makris, Dragana, 68100 Alexandroupolis, Greece; 5 Department of Internal Medicine, General Hospital of Larisa, Tsakalof 1, 41221 Larisa, Greece; 6 Department of Obstetrics and Gynecology, Democritus University of Thrace, 6th km Alexandroupolis–Makris, 68100 Alexandroupolis, Greece; 7 Department of Obstetrics and Gynecology, University General Hospital of Larissa, Mezourlo, 41110 Larissa, Greece; 8 Department of Pathology, Alexandra General Hospital of Athens, 11528 Athens, Greece; 9 Department of Obstetrics and Gynecology, General Hospital of Arta, 47100 Arta, Greece; 10 Department of Gynecology, Laiko General Hospital of Athens, Agiou Thoma 17, 11527 Athens, Greece

**Keywords:** polycythemia vera, portal vein, gestation, thrombosis, cesarean section

## Abstract

Polycythemia vera (PV) is one of the three main classic disorders of Philadelphia-negative myeloproliferative neoplasms (MPNs), with the other two being essential thrombocythemia (ET) and primary myelofibrosis (PMF). PV may develop (15%) in women of childbearing age (15–45 years), with an anticipated rate of roughly 0.3 per 100,000 people, although maintaining a male to female ratio predominance of about 2:1 and a peak prevalence in the sixth and seventh decades of life. Without always being presented with its actual clinical manifestations due to pregnancy itself, and most commonly due to iron deficiency, PV can be frequently missed and therefore belatedly diagnosed. We describe the case of a primipara woman in her 40s, without risk factors for thrombosis, who developed a portal vein occlusion 1.5 month postpartum after C-section and who had a delayed diagnosis of PV.

## 1. Introduction

This case report presents a primipara woman of 40 years of age who developed portal vein thrombosis 6 weeks after giving birth via C-section. Her medical history revealed no chronic illnesses, other than iron deficiency anemia (IDA) and occasional episodes of headache. During her hospitalization for the portal vein thrombosis, she had undergone full laboratory and imaging exams, which led to the delayed diagnosis of polycythemia vera (PV).

## 2. Case Report

A 40-year-old primipara woman presented to the hospital with fever and persistent abdominal pain of acute onset. She reported a history of an urgent C-section 45 days before, due to fetal heart rate decelerations and fetal distress. She delivered a healthy male neonate weighing 3000 gr. During her pregnancy, she received iron supplementation for a while, without being checked again for correction of anemia due to insufficient compliance of the patient. She also mentioned that due to her advanced age for pregnancy (40 years old), she had been given aspirin 100 mg/day from the early weeks of pregnancy till the 38th week, replaced after with LMWH (low molecular weight heparin) for 2 weeks. The day of delivery, her blood count was normal with mild anisocytosis. Her postoperative status was uncomplicated. During her hospitalization, the medical regimen she received had been 10 IU of oxytocin × 3/day, 1 amp of diclofenac/day and an antibiotic therapy with a second generation cephalosporine 1.5 gr/day for 3 days. She was discharged 4 days later, with over-the-counter LMWH for venous thrombosis prevention.

On her re-admission to the hospital, her clinical appearance and nutritional status were found to be normal, auscultation of the lungs appeared to be normal, and the abdomen was soft and sensitive with a palpable spleen. No focal neurological deficit was identified. When asked for her medical history, she only mentioned frequent headaches and iron deficiency anemia self-treated periodically with iron. A couple of hours before, she reported having visited a private laboratory and imaging clinical center where she performed a full blood count (FBC) and an abdominal ultrasound with the following results (Table 1).

### Upper Abdominal U/S Results

The liver was of a normal size and texture without focal or diffuse lesions on all four lobes. There was no dilatation of intrahepatic bile ducts, but a dilatation of the splenoportal axis with echogenic content and no blood flow was discovered. The gall bladder was also found to be normal while the pancreas was enlarged with heterogeneous consistency. Possible splenomegaly and partial ischemia of the spleen’s upper pole were diagnosed, after the spleen proved to be enlarged (14.7 × 6 cm), with a small area of reduced echogenicity in the middle and towards its upper pole, indicating the presence of thrombosis in the splenoportal axis.

Upon the woman’s arrival at the hospital, a new FBC, a CT (computerized tomography), an MRI (magnetic resonance imaging) scan and a gastroscopy were conducted (Table 2).

The CT scan findings were the following: a spleen of increased size with the presence of infarcts, accompanied by a splenoportal axis dilation with a subdense image throughout its course, in the main branch of the portal vein and the initial part of its left and right branches (extensive thrombosis). Moreover, an edematous texture of pancreas and distention of small intestine helices with congestion of the mesenteric vessels were also revealed. The MRI scan indicated the absence of blood flow in the upper mesenteric vein and throughout the splenoportal axis, suggesting extensive thrombosis of the above-mentioned vessels. The spleen, which was enlarged with extensive ischemic areas, indicated splenic vein infarcts, while the pancreas, the kidneys, the inferior vena cava and the hepatic veins were normal.

Additionally, the gastroscopy showed antral gastritis with two punctuate intramucosal infarcts in the bulb. Due to this thrombosis, a thrombophilia profile test was performed, with a negative result concerning any major acquired thrombophilic conditions, with the exemption of MTHFR C677T variant heterozygosity.

The patient was treated immediately with fondaparinux sodium 7500 IU 1 × 1 subcutaneously per day, acenocoumarol on gradual administration and meropenem 2gr × 3/day. Fifteen days later, she was discharged with instructions for continuation of the administered anticoagulant therapy. Her follow-up treatment was eventually altered to LMWH, 7 months after the use of acenocoumarol.

One year later, during a follow-up blood screen, abnormal liver laboratory values were found (Table 3).

A second admission to hospital was needed approximately two months later, with a diagnosis of portal hypertension and esophageal varices being confirmed. LMWH treatment was replaced again by acenocoumarol.

The FBC results were the following (Table 4).

**Figure 1:**
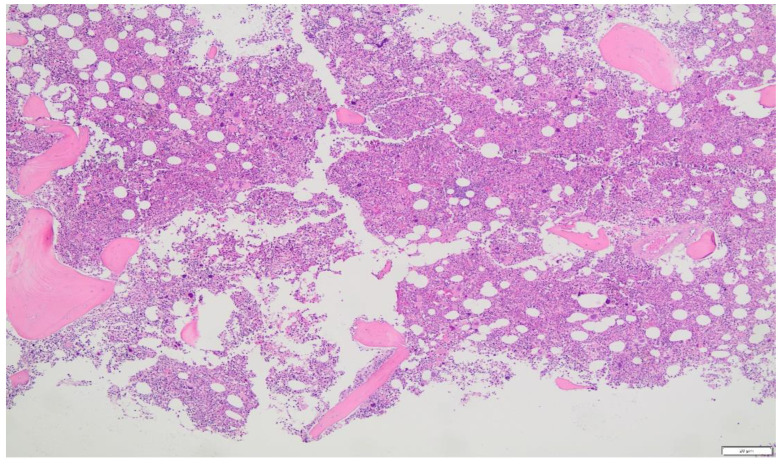

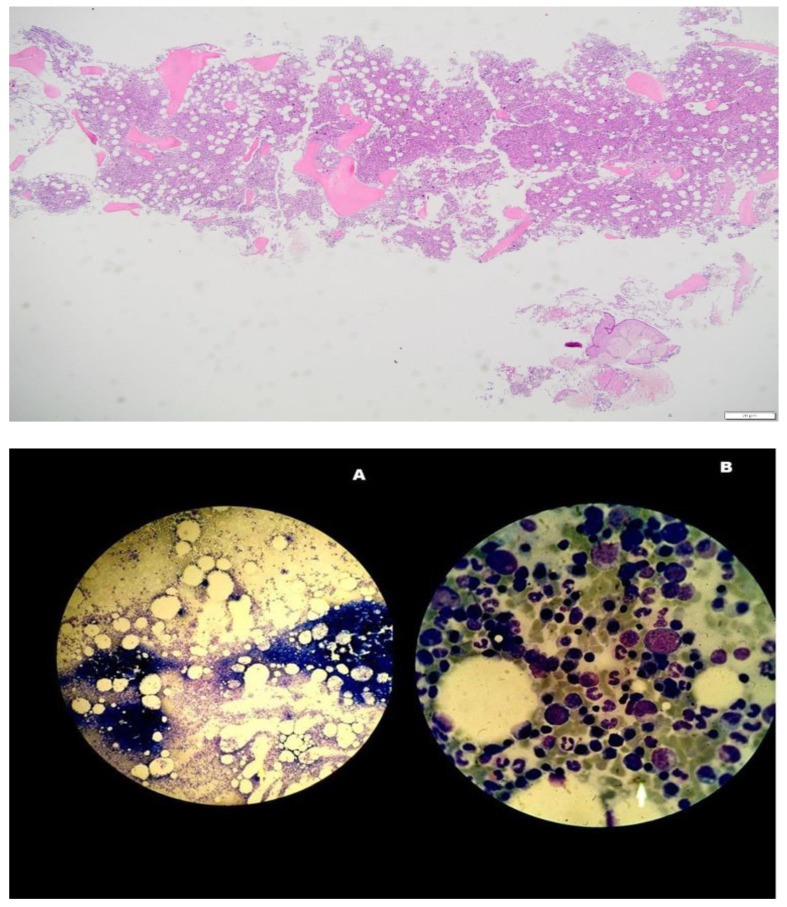
Panmyelosis in bone marrow aspirate supporting the diagnosis of polycythemia vera. ×2, ×4, ×20 (**A**) and ×100 (**B**) amplification by optical microscope.

Despite normal Hb and HCT levels, due to iron deficiency, elevated LDH and history of portal thrombosis, suspicion for MPN was established, for which JAK2V617F mutation was tested and found positive. Serum erythropoietin was tested low (1.8 mIU/mL), and a bone marrow biopsy was carried out at the same time, revealing panmyelosis (hypercellularity with trilineage growth), megakaryocytic growth with pleomorphic mature megakaryocytes and minor reticulin fibrosis. (Figure 1). When the patient was asked to present past records from the last 15 years, it was verified that she had chronic iron deficiency anemia corrected periodically with iron supplementation (Table 5).

Paradoxically, the patient had shown poor compliance for the standard routine exams during pregnancy. She had undergone blood screening tests only twice during her pregnancy: one on the 8th week of pregnancy and another one on the 26th week with the following results (Table 6).

## 3. Discussion

Polycythemia vera (PV), also known as Vasquez disease, is a chronic myeloproliferative disorder caused by a mutation in blood forming stem cells, which decreases the body’s ability to limit the production of blood cells, especially red blood cells. This causes an increase in red cell mass and blood’s viscosity, slowing down its movement through the veins and arteries. Thus, blood clots are formed, causing thrombosis of the splanchnic vessels such as hepatic, portal, superior mesenteric and splenic veins, individually or in combination [1,2,3].

PV, along with essential thrombocytosis, chronic myeloid leukemia and idiopathic myelofibrosis, constitute a group of stem cell disorders that lack the Philadelphia chromosome marker, demonstrating similar cytogenetic and molecular characteristics, and can be attributed to several genetic alterations [4]. In the majority of patients (97%), the mutation responsible for the abnormal proliferation of PV is the highly specific JAK2V617F, with the rest having the JAK2 exon 12 type, both activating the JAK-STAT pathway leading to a defective erythropoiesis and therefore polycythemia [5].

PV has a 0.8 to 7.8% thrombosis incidence rate, as well as an increased risk for bleeding, especially in patients with JAK2V617F mutation [3]. Because of the physical effects in the coagulation cascade that occur during pregnancy in order to prepare for delivery, gestation is a high-risk condition that may result in thrombosis [4]. In the event of co-existence of a myeloproliferative condition, the risk of thromboembolism is six times higher in pregnant women than in non-pregnant women [6]. Furthermore, pregnant women with PV demonstrate an increased likelihood for pregnancy-related complications, such as miscarriage, hypertensive disorders of pregnancy and intrauterine growth restriction as well as placental abruption [7].

A typical hematological profile of a patient with PV, according to 2016 WHO criteria [8], is the following:

## 4. Major Criteria

1. Red cell mass above average expected value by more than 25%, hemoglobin above 16.5 g/dL in males and above 16 g/dL in women or hematocrit above 49% in men and above 48% in women.
2. A BM biopsy demonstrating trilineage growth (panmyelosis) and hypercellularity, with strong erythroid, granulocytic and megakaryocytic proliferation and pleomorphic adult megakaryocytes (differences in size).
3. The existence of the JAK2V617F or exon 12 mutation.

## 5. Minor Criterion

1. A low level of erythropoietin (EPO) in the serum.

PV must either meet all three major criteria for diagnosis or the first two major criteria plus the minor one.

Patients may be clinically asymptomatic at the time of diagnosis and may only have isolated splenomegaly, erythrocytosis or thrombocytosis. WBC counts may be elevated. Increased hematocrit levels may be associated with a plethora of hyper viscosity symptoms, varying from headaches, blurred vision, erythromelalgia and peripheral ischemia with cyanosis to myocardial infarction, deep venous thrombosis or even portal, splenic and mesenteric vessel thrombosis when larger vessels are implicated.

PV in pregnancy is extremely rare, presenting a challenging diagnosis. A similar case report was recently published in which, however, PV was already diagnosed prior to the pregnancy, and as such, the management of the pregnancy followed the published recommendations [9]. The aforementioned case report highlights the difficulties in the diagnosis of such a condition and the need for a multidisciplinary approach in order to optimize the maternal and neonatal outcome.

Pregnancy itself, dilutional anemia (especially by the late second trimester) or other coexisting pathologies, most commonly iron deficiency anemia (IDA), is among the main causes.

It is important to note that the development of IDA occurs in three stages: pre-latent, latent and IDA stage. The muscle and liver cells’ ability to store iron gradually decreases during the pre-latent stage. Because the body manages the aged red blood cells by using iron from hemoglobin to fuel demands, these stockpiles can be exhausted without producing anemia. Although the hemoglobin levels remain within the normal range in the latent stage, the reserves are completely depleted. Reduced serum iron, ferritin levels and transferrin saturation are all characteristics of the IDA stage. Hemoglobin and hematocrit levels are poor in anemic patients but not until the remaining stores are fully gone. MCH and MCHC are likewise experiencing a decline. Since the red cell hemoglobin concentration (MCHC) is protected at the expense of red cell volume, RBCs shrink (microcytic erythrocytosis) in the presence of iron deficiency anemia of any form (MCV).

Despite having a high red cell mass, patients with PV originally have an artificially normal Hct level with microcytic RBC parameters due to an increase in RBC formation, even though they are iron deficient. A distinctive feature of PV is this confluence of results [3,10,11].

### 5.1. Some Recommendations about Management of PV in Pregnancy

There is a dearth of published information on MPN management during pregnancy. There are only some recommendations (Table 2) that are important to follow in order to protect pregnant women with PV from its most severe complications, such as thrombosis and, less frequently, hemorrhage [12]. These recommendations are based on our knowledge and information from patients with antiphospholipid syndrome and inherited thrombophilia. Pregnancy is generally not harmful in people with PV and other MPNs, and if it has already been confirmed, abortion is not strongly advised [13]. The most important factors in the management and diagnosis of PV are the prior obstetric history of the patient, which defines whether the pregnancy is of high risk (Table 3), and the disease status.

### 5.2. Important Issues Concerning Management of MPN and Pregnancy

#### 5.2.1. Planning Pregnancy and Preconception Phase

1. Avoid using teratogenic medications before getting pregnant.
2. Planning joint care with an MPN-experienced hematologist and consultant obstetrician.

#### 5.2.2. Pregnancy

1. At-risk pregnancy low-aspirin use (PV, CVRF, JAK2 positive MPN, recurrent abortions or stillbirths).
2. Strict venesection management of hematocrit (45%).
3. In high-risk circumstances, add IFN and aspirin.
4. Switch aspirin to LMWH two weeks before the anticipated delivery.
5. Until 24 weeks, FBC every 4 weeks. Τhen, two FBCs every week.
6. Blood pressure and a urine test at each appointment.
7. US scans performed at 12, 20, 26, 30, 34 and 38 weeks.
8. Uterine artery Doppler at 20 (+24 weeks if abnormal), i.e., bilateral high RI or notches:
  a. Increase the level of surveillance.
  b. Consider raising the LMWH dose.
  c. Include 1000 mg of vitamin C daily and 400 iu of vitamin E each day.
  d. An early birth before 38 weeks.

#### 5.2.3. Delivery

1. Once the patient enters labor, stop LMWH.
2. Do not administer LMWH within 12 h of delivery if you are having an elective cesarean section.

#### 5.2.4. Postpartum

1. LMWH at a preventative dose for six weeks after delivery.
2. Maintenance of the maternal hematocrit and platelet count at normal levels.

#### 5.2.5. Breastfeeding

1. Is not recommended for patients receiving cytoreductive treatment.

MPN: myeloproliferative neoplasms; INF: interferon; PV: polycythemia vera; CVRF: cardiovascular risk factor; LMWH: low molecular weight heparin.

High risk Pregnancy [12]

1. Mother’s history of arterial or vein thrombosis (whether pregnant or not).
2. Previous hemorrhages attributed to MPD (whether pregnant or not).
3. A previous pregnancy condition that MDP may have contributed to, e.g.,:
  a. Pregnancy losses in either the second or third trimester or three first trimesters.
  b. Birth weight below the gestational fifth centile.
  c. Stillbirth or intrauterine mortality (with no obvious other cause or evidence of placental dysfunction and growth restricted fetus).
  d. Considerable antepartum bleeding.
  e. Postpartum bleeding (requiring red cell transfusion).
  f. Severe pre-eclampsia (necessitating preterm delivery < 37 weeks).
  g. Any such condition developing during the index pregnancy.

2. A spike in platelet count to about 1500 × 109/L.

## 6. Conclusions

We described a case of a pregnancy with polycythemia vera undiagnosed over years, complicated postpartum with portal vein occlusion.

Concerning the patient, after the definitive diagnosis, she has undergone five endoscopic band ligations for esophageal varices, and she presented one episode of HIT (heparin-induced thrombocytopenia). The disease progressed, with palpable splenomegaly (ameliorated only for 4 months with the use of Ruxolitinib) and megakaryocytic proliferation with atypia accompanied by collagen fibrosis grade 3 in bone marrow biopsy.

Although it is hard to identify such pathology during pregnancy, a combination of a detailed medical history and a thorough monitoring of the pregnancy is strongly recommended under the joint care of obstetricians and hematologists.

## Figures and Tables

**Table 1:** Full blood count (FBC) and an abdominal ultrasound in a private laboratory and imaging clinical center.

WBC: 14,300	κ./μL	CRP: 117.6	mg/L
Hb: 11.5	g/dL	UREA: 15	mg/dL
Hct: 38	%	GLU: 92	mg/dL
PLT: 219	κ./μL	INR: 1.05	
SGOT: 30	U/L	CK-MB: 0.3	IU/L
SGPT: 36	U/L		
γ-gt: 45	IU/L		
LDH: 516	IU/L		
ALP: 134	U/L		
CPK: 56	IU/L		

**Table 2:** A new FBC, a CT (computerized tomography), an MRI (magnetic resonance imaging) scan and a gastroscopy in the hospital.

FBC	
WBC: 13.200	κ./μL
RBC: 4.75	κ./μL
Hb: 11.6	g/dL
Hct: 40.3	%
NEU: 76	%
MCV: 84.8	fl
MCH: 24.5	pg
MCHC: 28.9	g/dL
PLT: 260	κ./μL
SGOT: 27	U/L
SGPT: 41	U/L
Serum amylase: 35	IU/L

(Anisocytosis, leukocytosis and neutrophilia).

**Table 3:** A follow-up blood screen after one year.

WBC: 7900	κ./μL
RBC: 5160	κ./μL
Hct: 44.6	g/dL
Hb: 14	%
MCV: 88.4	fL
MCH: 26.8	pg
MCHC: 31	g/dL
PLT: 212	κ./μL
Fe: 53	ng/dL
Ferritin: 11	mg/mL
SGOT: 66	U/L
SGPT: 73	U/L
ALP: 591	U/L
LDH: 510	IU/L

(Anisocytosis and poikilocytosis).

**Table 4:** The FBC results.

WBC: 7900	κ./μL
RBC: 4490	κ./μL
Hct: 38.7	g/dL
Hb: 12	%
MCV: 86.2	fL
MCH: 26.8	pg
MCHC: 31	g/dL
PLT: 225	κ./μL
Fe: 37	mg/dL
Ferritin: 7.8	ng/dL
SGOT: 48	U/L
SGPT: 56	U/L
γ-GT: 90	IU/L
ALP: 239	U/L
LDH: 417	IU/L

(Hypochromia, anisocytosis and poikilocytosis).

**Table 5:** The patient’s past records.

15 Years Ago			15 Years Ago (3 Months Later)		
WBC: 5380	κ./μL		WBC: 6100	κ./μL	
RBC: 4960	κ./μL		RBC: 5200	κ./μL	
Hct: 44.1	%		Hct: 45	%	
Hb: 13.6	g/dL		Hb: 14.5	g/dL	
MCV: 88.9	fL		MCV: 86.3	fL	
MCHC: 30.9	pg		MCHC: 27.9	pg	
MCH: 27.9	g/dL		MCH: 32	g/dL	
PLT: 250	κ./μL		PLT: 261	κ./μL	
Fe: -	mg/dL		Fe: -	mg/dL	
Fer: 4.7	ng/mL		Fer: 6	ng/mL	
**12 Years Ago**		**5 Years Ago**		**4 Years Ago**	
WBC: 6000	κ./μL	WBC: 6400	κ./μL	WBC: 6400	κ./μL
RBC: 5450	κ./μL	RBC: 5160	κ./μL	RBC: 5040	κ./μL
Hct: 46.7	%	Hct: 42.8	%	Hct: 45.2	%
Hb: 15.4	g/dL	Hb: 13.7	g/dL	Hb: 14.2	g/dL
MCV: 85.7	fL	MCV: 82.9	fL	MCV: 89.7	fL
MCHC: 28	pg	MCHC: 26.6	pg	MCHC: 28	pg
MCH: 32	g/dL	MCH: 32	g/dL	MCH: 31.4	g/dL
PLT: 300	κ./μL	PLT: 349	κ./μL	PLT: 340	κ./μL
Fe: 49	mg/dL	Fe: 46	mg/dL	Fe: 45	mg/dL
Fer: 6.7	ng/mL	Fer: 8.9	ng/mL	Fer: 7.3	ng/mL
**3 Years Ago**					
WBC: 6340	κ./μL				
RBC: 5030	κ./μL				
Hct: 43.5	%				
Hb: 13.4	g/dL				
MCV: 86.5	fL				
MCHC: 26.6	pg				
MCH: 30.8	g/dL				
PLT: 400	κ./μL				
Fe: 41	mg/dL				
Fer: 4.5	ng/mL				

**Table 6:** Blood screening tests during pregnancy.

8th Week *		26th Week **	
WBC: 8930	κ./μL	WBC: 11,070	κ./μL
RBC: 4870	κ./μL	RBC: 4310	κ./μL
Hb: 14.2	g/dL	Hb: 12.2	g/dL
Hct: 44	%	Hct: 39.9	%
MCV: 90.3	fL	MCV: 92.6	fL
MCH: 29.2	pg	MCH: 28.3	pg
MCHC: 32	g/dL	MCHC: 30	g/dL
PLT: 341	κ./μL	PLT: 377	κ./μL
FE: 81	mg/dL	Fe: 75	mg/dL
Fer: 11	ng/mL	Fer: 3.9	ng/mL
GLU: 83	mg/dL	GLU: 84	mg/dL
SGOT: 20	U/L		
SGPT: 20	U/L		
UA: 5.1	mg/dL		
Cr: 0.61	mg/dL		
U: 25	mg/dL		

* With anisocytosis. ** With hypochromia and anisocytosis: iron deficiency anemia.

## Data Availability

Not applicable.
